# Prevalence and Positive Correlates of Posttraumatic Stress Disorder Symptoms among Chinese Patients with Hematological Malignancies: A Cross-Sectional Study

**DOI:** 10.1371/journal.pone.0145103

**Published:** 2015-12-15

**Authors:** Li Liu, Yi-Long Yang, Zi-Yue Wang, Hui Wu, Yang Wang, Lie Wang

**Affiliations:** Department of Social Medicine, School of Public Health, China Medical University, Shenyang, Liaoning, People’s Republic of China; North Shore Long Island Jewish Health System, UNITED STATES

## Abstract

**Purpose:**

Positive psychological constructs have been given increasing attention in research on the coping resources of cancer-related distresses. However, little research is available on posttraumatic stress disorder (PTSD) in patients with hematological malignancies. The purposes of this study were to assess the prevalence of PTSD symptoms and to explore the associations of perceived social support (PSS), hope, optimism and resilience with PTSD symptoms among Chinese patients with hematological malignancies.

**Methods:**

A cross-sectional study was conducted during the period from July 2013 through April 2014. A total of 225 inpatients with hematological malignancies, which were eligible for the study, completed the Post-traumatic Stress Checklist-Civilian Version, Multidimensional Scale of Perceived Social Support, Adult Hope Scale, Life Orientation Scale-Revised, and Resilience Scale. Hierarchical regression analysis was performed to explore the correlates of PTSD symptoms.

**Results:**

Overall, the prevalence of PTSD symptoms was 10.7%. Initially, PSS was negatively associated with PTSD symptoms (β = -0.248, *P* < 0.01). However, when positive psychological variables were added, optimism was negatively associated with PTSD symptoms (β = -0.452, *P* < 0.01), and gender had a significant effect on PTSD symptoms. Women were more vulnerable to these symptoms than men (β = 0.123, *P* < 0.05). When the analysis was performed separately by gender, only optimism showed a significantly negative association with PTSD symptoms in both men (β = -0.389, *P* < 0.01) and women (β = -0.493, *P* < 0.01).

**Conclusions:**

Some patients with hematological malignancies suffer from PTSD symptoms. The positive effects of PSS and optimism on PTSD symptoms suggest that an integrated approach to psychosocial intervention from both external and internal perspectives could have practical significance. Gender difference should be considered in developing potential interventions in reducing cancer-related PTSD symptoms.

## Introduction

Post-traumatic stress disorder (PTSD) is a psychiatric diagnosis characterized by the development of re-experiencing, avoidance and increased arousal symptoms following exposure to a traumatic event that threatens one’s psychological and/or physical integrity [1]. Conceptually, cancer differs from other known PTSD stressors (such as military combat, traffic accidents, rape, assault and natural disasters). However, cancer diagnosis and treatment, as well as its accompanying physical and psychosocial dysfunctions are considered to be traumatic stressors having the characteristics of repeated, long-term uncertainty and the capacity to initiate PTSD symptoms [2,3], a condition reflected in the current diagnostic criteria (the Diagnostic and Statistical Manual of Mental Disorders, Fourth Edition, DSM-IV, criterion A1, A2, B, C, D, E and F) [4].

PTSD symptoms are clinically relevant, because they are associated with impaired overall health, quality of life and survival in cancer patients [2,3,5,6]. The results of cancer-related PTSD studies have been inconsistent, with wide variations in prevalence rates, ranging from about 2% to 30%, and in potential correlates, likely due to differences in sample characteristics, definitions and diagnostic tools for PTSD across studies in different contexts [3,7]. Some demographic factors (age, gender, educational levels, economic status, etc.), clinical factors (type of cancer, stage at diagnosis/treatment, time since diagnosis/treatment, treatment type, etc.), and psychological and social factors (personality, coping style, social support, etc.) could be significantly associated with cancer-related PTSD symptoms [8–13]. However, little research is available on PTSD in patients with hematological malignancies, although a prevalence between 7.4% and 27% was reported in different types of hematological survivors [6,14–17]. Hematological malignancies, such as leukemia, lymphoma and multiple myeloma represent a kind of condition associated with considerable morbidity and mortality [18]. Although the overall outcomes of patients with hematological malignancies have been improved as a result of accurate diagnosis and both standardized and personalized treatment, most patients have to bear serious physiological, psychological and social consequences caused by the diseases. As a result, patients with hematological malignancies are at a high risk of suffering from PTSD [17,19].

Social support is an effective emotion regulator under conditions of traumatic stress that could contribute particularly to protection against cancer-related PTSD [9,16,19–22]. Social support refers to the perception and/or actuality that individuals are cared for and have assistance available in times of need from people in their supportive social networks [23]. However, little is known about the association between social support and PTSD symptoms among patients with hematological malignancies.

In addition to the positive roles of external social resources, with the rise of positive psychology, some internal psychological constructs (such as hope, optimism and resilience) have been given increasing attention in research on the coping resources of cancer-related distresses. As a multidimensional dynamic resource, hope is defined as confidence in relation to an uncertain future that provides the mental energy to choose and maintain a particular pathway through all stages of goal pursuit [24]. Optimism refers to a stable tendency to expect that good rather than bad things will happen, and that continuous efforts towards a desired goal will be influenced by the general tendency [25]. As a pattern of positive adaptation, resilience is expressed in one’s capacity to recover successfully from failure to attain a goal [26]. Such psychological constructs involve the positive coping styles of cancer patients, and they have been increasingly considered as important positive factors in the lives of these patients, including in helping them adjust to cancer and reduce psychological distresses [27–30]. However, to our best knowledge, studies on the potential effects of hope, optimism and resilience against PTSD symptoms are still rare among patients with hematological malignancies.

In light of the above concerns, and with the purpose of alleviating PTSD and its adverse effects among patients with hematological malignancies: firstly, the prevalence of PTSD symptoms was assessed; and secondly, the associations of perceived social support (PSS), positive psychological constructs (hope, optimism and resilience) with PTSD symptoms were examined. In addition, some demographic characteristics and clinical factors were considered as possible correlates of PTSD symptoms in this study.

## Materials and Methods

### Study design and sample

From July 2013 through April 2014, a cross-sectional study was conducted in consecutive inpatients with hematological malignancies from the Department of Hematology at Shengjing Hospital of China Medical University, which is an important service center for hematological malignancies in northeastern China. Patients with the following inclusion criteria were enrolled as potential subjects: (1) were at least 15 years old; (2) were diagnosed with hematological malignancies; (3) were aware of their own diagnoses, (4) had clear consciousness and cognition. Patients with the following criteria were excluded: (1) had other psychiatric or intellectual problems before cancer diagnoses; (2) had other types of cancers. All eligible patients were invited to participate in the study by their treating oncologists or attending physicians in charge.

The Committee on Human Experimentation of China Medical University reviewed and provided the ethics approval for this study, and the study procedures were in accordance with the ethical standards. Participation in the study was voluntary and anonymous. Participants were well-informed about the purpose and protocol of the study. Particularly, parent/guardian consent on behalf of minors under the age of 18 years was obtained in this study. After obtaining patients’ written consent, clinical data were collected from the medical record, and a set of questionnaires was distributed to the patients.

Initially, a total of 304 patients were enrolled. Forty-six patients refused to participate, and two patients had other types of cancers (ovarian cancer and lung cancer, respectively). Data with missing information concerning any item in the questionnaires were excluded from the final analysis. Thus, of the 256 eligible patients, 31 were excluded. In total, effective responses were received from 225 patients (87.9%) in this study (S1 Dataset).

### Measurement of PTSD symptoms

PTSD symptoms were measured by the Chinese version of the Posttraumatic Stress Checklist-Civilian Version (PCL-C) [31]. It consists of 17 items that reflect the DSM-IV symptom criteria of PTSD, including re-experiencing (items 1–5), avoidance/numbing (items 6–12) and increased arousal (items 13–17). Each item is scored on a 5-point Likert scale (from 1 “not at all” to 5 “extremely”) on the basis of how much the symptoms had disturbed them in tandem with the experiences of hematological malignancies and related treatment in the past month. The total score ranges from 17 to 85, with a higher total score indicating more severe PTSD symptoms. The cutoff method was used to identify PTSD, and those with total score ≥ 50 were classified as having PTSD [31].

The Chinese version of the PCL-C has been widely used in Chinese cancer patients with adequate reliability and validity [32,33]. In the present study, the Cronbach’s α for the total scale was 0.94, and the subscales produced the following internal consistency: re-experiencing, α = 0.86; avoidance, α = 0.86; and arousal, α = 0.87.

### Measurement of PSS

PSS was assessed by the Chinese version of the Multidimensional Scale of Perceived Social Support (MSPSS), which is a brief measurement of the respondent’s perception of the adequacy of the support he/she receives [34]. The 12-item MSPSS measures perceived support from three social relationships: family, friends and significant others. Items are scored on a 7-point Likert scale ranging from 1 “very strongly disagree” to 7 “very strongly agree” with possible scores ranging from 12 to 84, with a higher score indicating greater satisfaction with social support.

The MSPSS has good reliability and validity among various Chinese patients [35,36]. In the present study, the scale showed high internal reliability (Cronbach’s α coefficients were 0.84, 0.92, 0.84 and 0.93 for the family, friends and significant others subscales and the total scale, respectively).

### Measurement of hope

The Chinese version of the Adult Hope Scale (AHS) was used to assess the levels of hope in this study [37]. The AHS is a 12-item measure of an individual’s hope agency and hope pathways. The scale contains four agency and four pathway items, which are mixed with four filler items. Each item is scored on a 4-point Likert scale (from 1 “strongly disagree” to 4 “strongly agree”). Agency and pathway scores can be summed for a total hope score that ranges from 8 to 32. Higher total score indicates higher level of hope.

The Chinese AHS has been applied well to Chinese cancer patients [28,38]. In this study, Cronbach’s α values were calculated at 0.75, 0.79 and 0.84 for the agency subscale, pathway subscale and the total scale, respectively.

### Measurement of optimism

The Chinese version of the Life Orientation Scale-Revised (LOT-R) was used to measure optimism [39,40]. The scale consists of six target items and four filler items. There are three positive-worded items for positive general life expectations and three negative-worded items for negative general life expectations. Each item is scored on a 5-point Likert scale (from 1 “strongly disagree” to 5 “strongly agree”). The scores of positive-worded items and the inverted scores of negative-worded items can be summed for a total optimism score (ranging from 6 to 30). Higher total score indicates higher level of optimism.

The Chinese LOT-R has been demonstrated adequate reliability and validity in Chinese cancer patients [27,28]. In this study, the Cronbach’s α for this scale was 0.77.

### Measurement of resilience

The Chinese version of Resilience Scale (RS) was used to measure the level of patient’s resilience [41]. It consists of 14 items, and each item is rated on a 7-point Likert scale (from 1 “strongly disagree” to 7 “strongly agree”). For the factor structure of RS, personal competence consists of 10 items designed to measure self-reliance, independence, determination, invincibility, mastery, resourcefulness and perseverance, and acceptance of self and life consists of 4 items developed to measure adaptability, balance, flexibility and a balanced perspective on life. The total score ranges from 14 to 98, with higher score reflecting higher resilience.

The Chinese RS has been used in Chinese cancer patients with good reliability and validity [42]. In this study, Cronbach’s α values were 0.83, 0.74 and 0.89 for the personal competence subscale, acceptance of self and life subscale and the total scale, respectively.

### Demographic characteristics

The demographic characteristics of patients were collected, including age, gender, marital status, educational levels, household monthly income, place of residence and payment types. Age was collected as a continuous variable, and it was also divided into three groups: ≤ 35, 36–55 and ≥ 56 years. Marital status was categorized as married/cohabiting and single/divorced/widowed/separated. Educational levels were categorized as primary school or below, junior high school, senior high school, junior college, and university or above. Household monthly income (RMB) was categorized as ≤ 1000, 1001–2000, 2001–3000 and > 3000 yuan. The place of residence was categorized as urban or rural. Payment types were categorized as medical insurance or self-payment.

### Clinical variables

Clinical variables including disease types, time (number of months) since diagnosis, disease status, phase of chemotherapy and other chronic comorbidity were collected in this study. Disease types included acute leukemia, chronic leukemia, multiple myeloma, myelodysplastic syndrome, Hodgkin’s lymphoma and non-Hodgkin’s lymphoma. Time since diagnosis was collected as a continuous variable, which was also divided into four groups: ≤ 3, 4–6, 7–12 and > 12 months. Disease status was categorized as new onset, relapse/treatment failure and persistence. Phase of chemotherapy was categorized as none, inductive therapy, consolidation therapy and intensive therapy. If the respondents had ever been diagnosed with any common chronic disease (e.g., hypertension, hyperlipidemia, gastritis, arthritis, hepatic steatosis and diabetes), other chronic comorbidity was defined as “yes”.

### Statistical analysis

The demographic, clinical and psychological variables were described with median, mean, standard deviation (SD), number (*n*) and percentage (%) as appropriate. In this study, groups for which the response rate was less than 5% were combined for the categorical variables; as there were only 7 participants (3.6%) who had not received chemotherapy, this group was combined with the ‘‘inductive therapy” group. In addition, disease types were divided into two groups: leukemia (acute leukemia and chronic leukemia) and non-leukemia (multiple myeloma, myelodysplastic syndrome, Hodgkin’s lymphoma and non-Hodgkin’s lymphoma) due to the low response rate of certain specific types. The P-P-plot and K-S tests were used to verify the normal distribution of continuous variables. Group differences of continuous variables were examined using independent sample *t*-test or one way analysis of variance (ANOVA). Pearson’s chi-square (*χ* ^2^) test was used to compare differences in categorical variables. Pearson’s correlation was used to examine correlations among continuous variables. Hierarchical regression analysis was used to explore the associations of PSS, hope, optimism and resilience with PTSD symptoms. Data including *F* value, *R* ^2^, *R* ^2^-changes (Δ*R* ^2^), standardized regression coefficient (β) and *P* value for each step in the regression model were reported. All study variables were centralized before analysis to account for differences in scale scores. Moreover, multi-collinearity was checked by tolerance and variance inflation factor (VIF). All analyses were conducted using SPSS for Windows, Ver. 13.0, with two-tailed probability value of < 0.05 considered to be statistically significant.

## Results

### Descriptive statistics

Demographic and clinical variables of participants and group differences of PTSD symptoms are shown in Table 1. The participants were in the age range of 15–83 years (Mean ± SD: 45.30 ± 16.02). Of these participants, 52.0% (117) were men, 78.7% (177) were married/cohabiting, and 33.8% (76) received a junior college or above education. Eighty-seven participants (38.7%) had a household monthly income level of ≤ 1000 yuan RMB, 142 (63.1%) participants lived in an urban area, and 53 (23.6%) had to pay for medical care themselves. With regard to clinical variables, 149 (66.2%) participants were leukemia survivors, the mean time since diagnosis was 4.50 months (range: 1–85 months), 118 (52.4%) had a persistent disease status, 108 (48.0%) were inpatients at the phase of consolidation treatment, and 129 (57.3%) reported that they had at least one other chronic condition. Statistically significant relationships between PTSD symptoms and demographic and clinical variables were not found in this study.

**Table 1:** Demographic and clinical variables of participants in relation to PTSD symptoms.

			PTSD symptoms			
Demographic variables	*n*	%	Mean	SD	*F*/*t* value	*P* value
**Age (years)**					0.42	0.66
≤ 35	72	32.0	32.64	12.64		
36–55	84	37.3	32.81	11.51		
≥ 56	69	30.7	31.12	12.64		
Mean (SD)	45.30 (16.02)					
Median (Range)	47.00 (15–83)					
**Gender**					0.05	0.96
Men	117	52.0	32.27	11.77		
Women	108	48.0	32.20	12.71		
**Marital status**					0.07	0.94
Married/cohabiting	177	78.7	32.35	11.76		
Single/divorced/widowed/separated	48	21.3	32.21	12.35		
**Educational level**					2.34	0.06
Primary school or below	20	8.9	31.98	14.19		
Junior high school	88	39.1	34.18	12.68		
Senior high school	41	18.2	30.88	10.54		
Junior college	34	15.1	27.20	8.70		
University or above	42	18.7	33.69	13.26		
**Household monthly income (yuan)**					0.56	0.64
≤ 1000	87	38.7	33.45	12.20		
1001–2000	66	29.3	30.90	11.67		
2001–3000	36	16.0	32.04	10.71		
≥ 3000	36	16.0	31.96	14.56		
**Place of residence**					1.36	0.18
Urban	142	63.1	31.39	12.11		
Rural	83	36.9	33.68	12.29		
**Payment type**					0.48	0.63
Medical insurance	172	76.4	32.02	12.21		
Self-payment	53	23.6	32.94	12.24		
**Clinical variables**						
**Disease type**					1.67	0.10
Leukemia	149	66.2	33.21	12.32		
Acute leukemia	136	60.4				
Chronic leukemia	13	5.8				
Non-leukemia	76	33.8	30.34	11.81		
Multiple myeloma	27	12.0				
Myelodysplastic syndrome	2	0.9				
Non-Hodgkin’s lymphoma	43	19.1				
Hodgkin’s lymphoma	4	1.8				
**Time since diagnosis (months)**					0.71	0.55
≤ 3	101	44.9	31.36	12.01		
4–6	42	18.7	33.20	11.31		
7–12	31	13.8	30.97	13.34		
> 12	31	22.7	33.95	12.66		
Mean (SD)^a^	4.50 (3.52)					
Median (Range)	4.00 (1–85)					
**Disease status**					1.41	0.25
New onset	49	21.8	30.97	11.83		
Relapse/treatment failure	58	25.8	34.50	12.82		
Persistence	118	52.4	31.65	11.99		
**Phase of chemotherapy**					2.07	0.13
None or inductive therapy	89	39.6	30.22	11.63		
Consolidation therapy	108	48.0	33.71	11.72		
Intensive therapy	28	12.4	32.99	15.08		
**Other chronic comorbidity**					0.12	0.91
Yes	96	42.7	32.35	12.30		
No	129	57.3	32.15	12.17		

PTSD = posttraumatic stress disorder, SD = standard deviation.

^a^The mean is logarithmic.

The levels of PTSD symptoms, PSS, hope, optimism and resilience are provided in Table 2. The mean score of PTSD symptoms was 32.24 (SD = 12.20), which ranged from 17 to 76. Based on the cut-off value of 50, the prevalence of PTSD symptoms in patients with hematological malignancies was 10.7%. The mean scores were 66.63 (SD = 13.20), 21.77 (SD = 5.20), 21.49 (SD = 3.95) and 70.66 (SD = 15.94) for PSS, hope, optimism and resilience, respectively.

**Table 2:** Descriptive statistics for PTSD symptoms, PSS, hope, optimism and resilience.

Variables	Mean	SD	Range	*n* (%)
**PTSD symptoms**	32.24	12.20	17–76	
Scores ≥ 50				24 (10.7)
**PSS**	66.63	13.20	26–84	
**Hope**	21.77	5.20	8–32	
**Optimism**	21.49	3.95	8–30	
**Resilience**	70.66	15.94	25–98	

PTSD = posttraumatic stress disorder, PSS = perceived social support, SD = standard deviation.

### Correlations among PSS, hope, optimism, resilience and PTSD symptoms

Pearson’s correlation coefficients among PSS, hope, optimism, resilience and PTSD symptoms are shown in Table 3. The level of PTSD symptoms was negatively correlated to PSS (*r* = -0.246, *P* < 0.01) and the three positive psychological variables (hope: *r* = -0.194, *P* < 0.01; optimism: *r* = -0.464, *P* < 0.01; resilience: *r* = -0.247, *P* < 0.01). PSS was positively correlated to hope (*r* = 0.396, *P* < 0.01), optimism (*r* = 0.573, *P* < 0.01) and resilience (*r* = 0.383, *P* < 0.01), respectively. In addition, there were significantly positive correlations among hope, optimism and resilience.

**Table 3:** Correlations among PTSD symptoms, PSS, hope, optimism and resilience.

Variables	1	2	3	4
**1. PTSD symptoms**	–			
**2. PSS**	-0.246**	–		
**3. Hope**	-0.194**	0.383**	–	
**4. Optimism**	-0.464**	0.351**	0.389**	–
**5. Resilience**	-0.247**	0.573**	0.519**	0.425**

PTSD = posttraumatic stress disorder, PSS = perceived social support.

**P < 0.01.

### Hierarchical regression results predicting PTSD symptoms

Univariate analyses failed to find any significant associations of demographic and clinical variables with PTSD symptoms. Age and gender (gender was coded as a dummy variable) were considered as control variables and added to Block 1. As shown in Table 4, PSS was significantly and negatively associated with PTSD symptoms (β = -0.248, *P* < 0.01), and it accounted for 6.4% of the variance in the prediction of PTSD symptoms in Block 2. Hope, optimism and resilience together accounted for an additional 17.4% of the variance in the prediction of PTSD symptoms in Block 3. However, only optimism showed a significant and negative association with PTSD symptoms (β = -0.452, *P* < 0.01). Tolerance (range: 0.53–0.95) and VIF (range: 1.06–1.88) indicated that multi-collinearity could be accepted in the regression model. Gender had a significant effect on PTSD symptoms when positive psychological variables were entered into the model (Block 3). Women were more vulnerable to PTSD symptoms than men (β = 0.123, *P* < 0.05). Therefore, two hierarchical regression analyses were conducted to explore potential gender differences in these associations.

**Table 4:** Hierarchical regression for exploring the positive correlates of PTSD symptoms.

Variables	Block 1 (β)	Block 2 (β)	Block 3 (β)
**Age**	-0.076	-0.049	-0.031
**Gender**	0.006	0.044	0.123*
**PSS**		-0.248**	-0.097
**Hope**			0.024
**Optimism**			-0.452**
**Resilience**			-0.029
***F***	0.636	5.078**	11.370**
***R*** ^**2**^	0.006	0.064	0.238
**Δ*R*** ^**2**^	0.006	0.059**	0.174**

PTSD = posttraumatic stress disorder, PSS = perceived social support.

Gender: women vs. men.

*P < 0.05,

**P < 0.01.

There was no significant difference in demographic and clinical variables between male and female patients. As shown in Table 5, the levels of PSS, optimism and resilience of women were significantly higher than those of men. In men, PSS was significantly associated with PTSD symptoms (β = -0.322, *P* < 0.01), and accounted for 11.2% of the variance in the prediction of PTSD symptoms in Block 2. However, the effect of PSS on PTSD symptoms was not significant in women. Hope, optimism and resilience together accounted for an additional 14.9% and 21.0% of the variance in the prediction of PTSD symptoms in men and women in Block 3, respectively. Moreover, only optimism had a significant and negative association with PTSD symptoms in both men (β = -0.389, *P* < 0.01) and women (β = -0.493, *P* < 0.01). Tolerance (range: 0.49–0.96) and VIF (range: 1.04–2.04) indicated that multi-collinearity could be accepted in the two regression models.

**Table 5:** Hierarchical regression for exploring the positive correlates of PTSD symptoms in men and women, respectively.

	Variables	Mean (SD)	Block 1 (β)	Block 2 (β)	Block 3 (β)
**Men**					
*n* = 117	**Age**	43.46 (16.39)	-0.091	-0.076	-0.036
	**PSS**	64.50 (13.49)		-0.322**	-0.096
	**Hope**	21.40 (5.40)			0.005
	**Optimism**	20.63 (4.14)			-0.389**
	**Resilience**	67.96 (16.48)			-0.116
	***F***		0.966	7.171**	7.822**
	***R*** ^**2**^		0.008	0.112	0.261
	**Δ*R*** ^**2**^		0.008	0.103**	0.149**
**Women**					
*n* = 108	**Age**	47.30 (15.44)	-0.059	-0.028	-0.021
	**PSS**	68.94* (12.53)		-0.163	-0.103
	**Hope**	22.18 (4.97)			0.046
	**Optimism**	22.43** (3.53)			-0.493**
	**Resilience**	73.59** (14.86)			0.059
	***F***		0.368	1.573	6.426**
	***R*** ^**2**^		0.003	0.029	0.240
	**Δ*R*** ^**2**^		0.003	0.026**	0.210**

PTSD = posttraumatic stress disorder, PSS = perceived social support, SD = standard deviation.

*P < 0.05,

**P < 0.01.

## Discussion

In the present study on PTSD symptoms in patients with hematological malignancies, based upon the scoring method (PCL-C scored 50 and above), 10.7% of our sample (10.3% in men; 11.1% in women) reported PTSD symptoms. Using the same diagnostic tool and conservative cutoff score, cancer-related PTSD prevalence was determined in the range between 1.9% and 12% in previous studies of patients with breast cancers, which decreased over time since diagnosis or treatment [5,8,43–49]. The prevalence of PTSD symptoms in our sample was similar to that in recently diagnosed and treated cancers patients [43,44], but higher than that of patients with gynecologic cancer (6.08%) in China [33]. Using the Posttraumatic Diagnostic Scale (PDS), 13% of the Hodgkin’s lymphoma survivors were diagnosed with PTSD [14]. There were 18% of long-term survivors of Hodgkin’s and non-Hodgkin’s lymphoma who met the diagnostic criteria of the PTSD inventory in Israel [15]. The bias-adjusted prevalence of PTSD was similar using the cutoff score ≥ 44 of PCL-C (7.4%) and symptom cluster (7.9%) methods in long-term non-Hodgkin’s lymphoma survivors [16]. Fifty-four of the 200 acute leukemia participants (27%) who scored ≥ 40 on the Stanford Acute Stress Reaction Questionnaire (SASRQ) demonstrated PTSD [6]. Among patients with different hematological malignancies, 17% met diagnostic criteria for current PTSD using PCL-C symptom cluster method [17]. In fact, based upon the results of currently available studies, clear and reasonable comparisons with the present study could not be carried out, because the knowledge about PTSD in hematological malignancies is relatively limited to our best knowledge, and there were differences in the measurement tools and diagnostic methods used across studies [6,14–17]. Consequently, the present study demonstrated that some patients with hematological malignancies suffered from clinically significant symptoms of posttraumatic stress; in fact, understanding and addressing patients’ existing needs would have more practical significance than pathologizing them.

As shown in the regression analysis, when the positive psychological constructs were considered, the significant effect of gender on PTSD symptoms was revealed in this study. This result indicated that when men and women possessed the same level of positive psychological resources, women were more prone to suffer from PTSD symptoms. Fortunately, we found that the levels of optimism and resilience of women were significantly higher than those of men, which would help them cope with PTSD symptoms. Some previous studies have identified the gender difference in responses to various traumatic events [50,51]. One possible reason is that there are significant differences in cognizing and dealing with emergency events based upon different personalities, psychological states, and social roles and functions between both genders [51–53]. However, the other demographic factors considered in this study were not significantly associated with PCL-C scores in both univariate and multivariate analyses. Cancer-specific variables such as disease types, clinical stages and treatments were seldom significantly associated with the PTSD symptoms of cancer survivors as reported previously [7], which was further confirmed by the present study.

This finding, consistent with other research showing that PSS was significantly associated with PTSD symptoms, showed that supportive environments were correlated with less psychological distress in cancer patients [9,16,19,21,22]. Supportive social network can help individuals cope with various stressful events and serve as a buffer against their negative health impacts. PSS might be particularly important for patients with hematological malignancies, because the illness, treatment and prolonged hospitalization necessitate supportive relationships between patients and their environments. Interestingly, the significant effect of PSS on PTSD symptoms was only shown in men, leading to gender dependence in the study. When faced with traumatic events, the level of social support of women is usually higher than that of men due to the deviation in initiative seeking of medical and social supports [54,55]. In Chinese culture, men often possess more diverse social roles and functions compared with women, while the later are more confined to family context [56,57]. The social isolation and dysfunction caused by cancer contribute to patients’ loneliness and helplessness, especially for male patients. Thus, functional support from various social relationships could be more valuable to men than to women for coping with PTSD symptoms.

The results from univariate analyses showed that the three positive psychological variables were negatively correlated with PTSD score, but only optimism had a significant effect on PTSD symptoms in the multivariate regression analyses in both genders. In fact, these positive psychological constructs are not only correlated with each other, but also have causal relationships based on the research on positive psychology, cognitive and emotional theories. Optimism contains cognition, emotion and motivation components, which can directly or indirectly increase individuals’ hope level in a positive way [58]. Hope not only reflects individuals’ determination to achieve their goals, but also involves the faith to develop a plan to succeed and to identify an effective way to implement this plan. People with higher hope levels recover more easily from frustration, so that they are more resilient [59]. In essence, optimism is a personality trait, and at its theoretical core are positive expectations about future events. Moreover, optimism can be viewed as an explanatory style, which is associated with a positive outcome outlook or attribution of success [60]. When individuals suffer from serious traumatic stressors, optimism can instantly play a positive role in maintaining mental health. However, hope and resilience refer to state-like positive psychological capabilities, which have undergone extensive theory-building and research. Compared with optimism, more external resources are needed to foster the positive roles of hope and resilience, such as knowledge and skills, life experiences, social networks and economic status [61]. In addition, it may take a long time for state-like capabilities like hope and resilience to take effect because the generation and development of both require the processes of cognition, emotion and motivation to work against the traumatic stressors from hematological malignancies. In the present study, the mean time since diagnosis of this sample was 4.50 months. Hope and resilience could not yet have positive effects on PTSD symptoms. Future research is needed to check whether or not the length of time since diagnosis or treatment moderates the relationships between positive psychological constructs and PTSD symptoms. Moreover, hope, optimism and resilience together accounted for an additional 21.0% of the variance in the prediction of PTSD symptoms in women and 14.9% in men. As mentioned above, women’s optimism and resilience levels were significantly higher than men. It suggested that close attention should be paid to gender difference when the intervention strategies and measures of PTSD are developed and implemented. When positive psychological variables were introduced, the effect of PSS disappeared. This might be due to the effect of dispositional optimism on the perception of social support [62]. It also indicates that internal psychological resources could be more effective for dealing with traumatic events than external coping resources.

These findings lead us to believe that both optimism and PSS are important to alleviate PTSD symptoms successfully in patients with hematological malignancies. Although optimism was measured as a personality trait in the present study, it could also be considered as a positive explanatory style that can be intervened and developed. Previous studies developed the psychosocial interventions to enhance the level of optimism in cancer patients [63,64]. The extent to which specific or routine specialized psychological interventions may reduce PTSD symptoms and other distresses requires further study in these patients as well as in other fields of oncology. The positive effect of optimism was stronger than that of PSS in this study, but specific and adequate social supports from various relationships may be valuable in helping these patients further reduce the psychological distresses of hematological malignancies, especially for men.

There are several limitations to the present study. Firstly, it was conducted at a single regional treatment center, and no differences in demographic characteristics and clinical factors were determined between participants and those who refused participation. Further multi-center and large sample size research could provide a good representation of patients with hematological malignancies and contribute to the generalization of our findings. Secondly, the cross-sectional design of this study was unable to assess the causal relationships among study variables, and only provided a snapshot of the PTSD symptoms that were experienced by the patients at the time of the survey. The findings would need to be confirmed in longitudinal research. Thirdly, study variables were mainly detected using self-report measures. There was a possibility of recall and reporting bias, and associations among these variables might be influenced. Some effective process control measures were carried out to minimize the potential common-method bias. Finally, further studies are needed to examine the reasons for the gender difference showed in the present study, and whether the difference exists in other types of cancer patients and cultural contexts.

## Conclusions

Some patients with hematological malignancies suffer from PTSD symptoms. The associations of PTSD symptoms with PSS and optimism suggest that an integrated approach to psychosocial intervention from both external and internal perspectives could have practical significance. Optimism could have an effect on coping resources in reducing PTSD symptoms for both genders. Gender difference should be taken into account when potential intervention is developed to reduce the psychological distresses of hematological malignancies as well as any other types of cancers.

## Supporting Information

S1 Dataset Supporting dataset. The supporting dataset includes the data underlying our findings in this study.
(XLS)
